# Review: Advanced Atomic Force Microscopy Modes for Biomedical Research

**DOI:** 10.3390/bios12121116

**Published:** 2022-12-02

**Authors:** Fangzhou Xia, Kamal Youcef-Toumi

**Affiliations:** Mechatronics Research Lab, Massachusetts Institute of Technology, Cambridge, MA 02139, USA

**Keywords:** atomic force microscopy, nanotechnology, mechanobiology, high-speed imaging, material property mapping, nano-manipulation, biomedical research

## Abstract

Visualization of biomedical samples in their native environments at the microscopic scale is crucial for studying fundamental principles and discovering biomedical systems with complex interaction. The study of dynamic biological processes requires a microscope system with multiple modalities, high spatial/temporal resolution, large imaging ranges, versatile imaging environments and ideally in-situ manipulation capabilities. Recent development of new Atomic Force Microscopy (AFM) capabilities has made it such a powerful tool for biological and biomedical research. This review introduces novel AFM functionalities including high-speed imaging for dynamic process visualization, mechanobiology with force spectroscopy, molecular species characterization, and AFM nano-manipulation. These capabilities enable many new possibilities for novel scientific research and allow scientists to observe and explore processes at the nanoscale like never before. Selected application examples from recent studies are provided to demonstrate the effectiveness of these AFM techniques.

## 1. Introduction

Many fundamental discoveries from biomedical research arise from studies at the microscopic scale. The human body is composed of trillions of cells, which interact with trillions of microscopic living organisms like bacteria and virus every day. Microorganisms are ubiquitous and typically too small to be seen by the naked eye. To study these microscopic organisms and their interactions, the development of microscopy instruments has become essential for scientists to conduct fundamental research.

The study of complex samples in biomedical research can benefit significantly from advanced microscopy instruments. Taking the virus-cell interaction as an example, viruses can infect all types of life forms, from animals and plants to microorganisms. Although viruses exhibit many modes of infection, all of them are parasites that must infiltrate hosts in order to replicate. Most viruses are enveloped in a membrane such as the coronavirus. The presence of this envelope has a profound influence on the attributes of the virus, especially when encountering the plasma membrane of the host cell. Virus-receptor interactions play a key role in viral internalization. The viral attachment proteins can be viewed as the “key” that unlocks host cells by interacting with the “lock”, which are the receptors on the host cell surface. These lock-and-key interactions results in the formation of pores that allow passage of the viral core into the interior of the cell. To better understand these interactions, microscopic visualization techniques that can resolve mechanical properties and molecular species with high-temporal and high-spatial resolution in liquid are needed.

**Figure 1:**
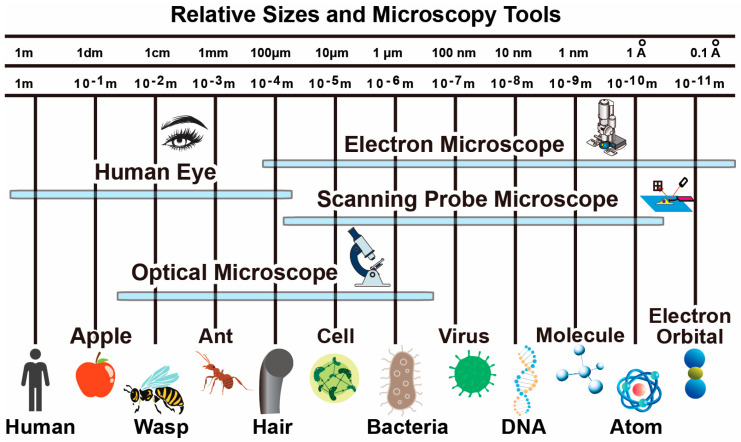
Typical size of biological objects and resolution capabilities of microscopy techniques.

Microscopy techniques relevant to biomedical research primarily includes optical microscopy, (cryo)-electron microscopy and scanning probe microscopy. They corresponds to three types of interactions with the sample including electromagnetic radiation (light at different wavelength), electron scattering, and mechanical force. As shown in Figure 1, different types of microscopy techniques have their own resolution capabilities, target modalities, operation environments and suitable sample preparation steps. Depending on the application requirements, corresponding techniques can be selected. In many demanding applications, multiple techniques are combined for in-situ characterization of samples. Note that many other microscopy techniques are also available such as X-ray crystallography and nuclear magnetic resonance imaging, which are more relevant for studies in other fields such as physics and material science and therefore not discussed in details here.

Optical microscopy is widely used by microbiologists to study micrometer scale objects. Depending on the type of light source configurations, optical microscopy can in general be classified into bright field, dark field and fluorescence. Bright field microscopy is the most widely used tool to image biological samples typically stained and fixed on glass slides. In this mode, the background is bright whereas the sample specimen is dark. To improve the spatial resolution, dark field microscopy with more sophisticated optical setup is utilized where the sample is bright and the background is dark. The resolution is limited by diffraction as governed by the Rayleigh criterion. Fluorescence microscopy can further improve spatial resolution by highlighting target structure with fluorescence dye to emit light at various wavelength in response to ultra-violet light source. Better contrast can be obtained as the neighboring structure does not emit light and multiple colors can be obtained.

Even with fluorescence microscopy, the spatial resolution is still limited to above one hundred nanometers. This resolution allows the study of cells but prevents the study of smaller samples such as viruses and DNA molecules. Moreover, light-based optical techniques cannot directly obtain mechanical property information, such as stiffness and adhesion. Therefore, another microscopy instrument is needed to overcome such limitations.

Electron microscopy is an alternative technique that can create subnanometer resolution images. Electron microscopes create images by shining a beam of electrons onto the sample and measure the properties of back-scattered, secondary or transmitted electrons to create contrast images. It is widely used in nanotechnology research on (semi)conductive samples. The main limitation for its application on biological sample is the required operation environment in vacuum and sample coating for conductivity. Biological samples can easily be damaged during the process required before imaging. To better preserve the sample structure integrity, cooling the aqueous biological sample down to cryogenic temperature to form amorphous ice allows better imaging of the biological sample structure down to near-atomic resolution. However, such processing forbids the observation of dynamic changes in the biological samples and also cannot provide mechanical property information.

Atomic Force Microscopy (AFM), a member of the Scanning Probe Microscopy (SPM) family, is a great complementing tool to resolve the aforementioned limitations. First, AFM is a versatile tool that can operate in ambient air, high vacuum and liquid environments, which makes it an ideal imaging technique for biomedical research. Biological samples can be imaged in buffer solution by AFM, which is helpful to preserve specimens in their native states. Second, an AFM in its basic form can obtain 3D topography information [1] by scanning a cantilever probe over the sample surface. AFM typically has subnanometer out-of-plane resolution and in-plane resolution on the order of several nanometers. It can resolve DNA double helix [2] or even atomistic events at the atomic lattice step edges in the right conditions [3]. The probe-sample interactions can be of various nature and result in the deflection change of a micro-cantilever. As a result, the mechanical interaction between the probe and the sample can also be easily captured to create material property mappings. Third, AFM probes used for imaging also offer a more direct way to mechanically manipulate the sample compared to optical tweezers and electron beam based fabrication (e.g., lithography, welding, evaporation, etc.). These characteristics of AFM make it an attractive tool for biomedical applications. Scanning Tunneling Microscope (STM) is another type of SPM system using a rigid conductive tip and the tunneling current to image conductive samples with atomic resolution. It is used more often in material science research instead of biomedical research due to more stringent requirements of the sample and imaging environments. A summary of the aforementioned microscopy categories is summarized in Table 1. Notice that values provided are for typical systems while specialized implementation can significantly change the characteristics of the instrument. For example, in high-speed AFM imaging to be discussed in details later, the frame rate can be significantly increased at the cost of a smaller imaging area.

Compared to optical and electron microscopy techniques, conventional AFMs also have several limitations. First, the AFM imaging speed is relatively slower due to the serial mechanical scanning process of the cantilever probe. Second, to avoid damage to the fragile biological samples, AFM operation is more complicated as it involves mode selection and controller parameter tuning to ensure good imaging quality. Third, AFM cannot sense electromagnetic spectrum directly in its native form (e.g., light color), which may contain important material property information. On the microscopic scale, color can be considered as the absorption/reflection spectrum of the sample material across a wide range of wavelength. A pair of electromagnetic wave emission and detection source is typically required for the measurement of such spectrum, which is not natively available in a conventional AFM setup. Consequently, it is challenging to use conventional AFMs in its basic form to meet the challenging demands in biomedical research. Fortunately, the AFM technique has evolved rapidly to realize new capabilities and overcome existing limitations based on experimental needs by proper modification of the AFM system and combination with other techniques. In this paper, we review recently developed advanced AFM capabilities that are useful for biomedical research. Both principle level discussion of the instrument development and application examples are provided for researchers to better understand and make use of these new AFM capabilities.

The paper is organized as the following. First, the basic design and operation principles of AFMs are introduced. Second, typical modes of operation and experimental considerations for biomedical samples are covered. Third, three categories of specialized AFM imaging capabilities are reviewed, including high-speed dynamic process visualization, mechanobiology studies, and molecular species characterization. Fourth, the ability to use the AFM as a nano-manipulation tool with in-situ characterization capability is discussed for applications such as cell manipulation and substance injection. In the end, additional biomedical application examples are presented, followed by a conclusion on future directions of AFM capability development for biomedical research.

## 2. AFM Basic Principles

**Figure 2:**
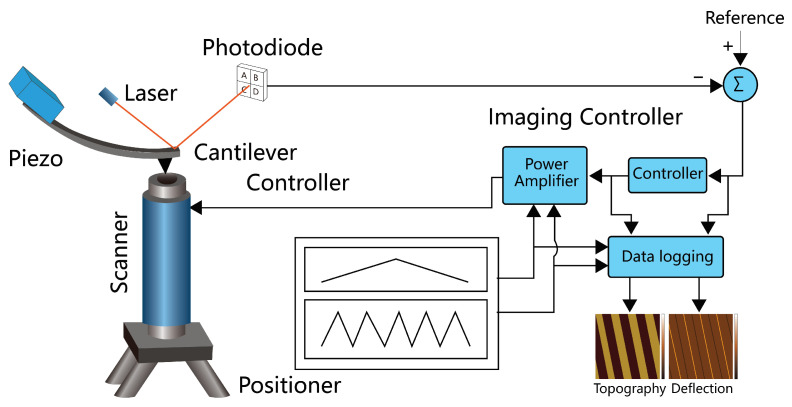
Principle illustration of a conventional AFM including a cantilever probe with optical beam deflection sensor and piezo-acoustic resonance excitation, a nanopositioning system, and an imaging motion controller.

A typical AFM can create 3D topography images with a range over hundreds of microns at subnanometer resolution. The out-of-plane resolution of AFM can reliable resolve single atomic layer steps of graphene, which is around 0.35 nm. The system vibration level and performance of the nano-positioning system is the main limiting factor of the out-of-plane resolution. The in-plane resolution of AFM is mostly limited by the probe tip radius and modes of operation with a typical value on the order of several nanometers. In specialized conditions, subnanometer resolution is possible [3], while in other cases where metallic tip coating is applied, the resolution is limited to tens of nanometers. To realize nanoscale topography imaging capabilities, a conventional AFM is primarily composed of three subsystems. They are the cantilever probe, the nano-positioning system, and controller electronics, as shown in Figure 2.

### 2.1. Cantilever Probe

A flexible microcantilever probe with a tip, sensors and actuators is used to interact with the sample. A typical probe is composed of a base support chip, a microcantilever, and a sharp tip. Probes are typically made with silicon, silicon dioxide or silicon nitride material created through nanofabrication processes. In most cases, the cantilever deflection is measured using an optical beam deflection system to achieve sub-nanometer resolution. A laser source and a quadrature photodetector work together to measure the free end angle change of the cantilever, which is proportional to the deflection at small angles. A piezo actuator can be used to acoustically excite the resonance of the cantilever for dynamic mode operation. Going beyond conventional implementation, additional sensing methods have also been investigated including astigmatic detection, interferometry, optomechanical sensing, piezoresistivity, and piezoelectricity. Alternative actuation methods include piezoelectric, photothermal, thermomechanical, electrostatic, and electromagnetic principles. A more detailed review of these principles is available in [4].

For advanced applications, both the tip and the micro-cantilever of the AFM probe can be upgraded. For example, the tip geometry and material can be modified to image high-aspect ratio samples with sharp tip, perform nano-indentation using spherical tip, improve laser detection performance with reflective gold coating or create electrical property mapping with conductive tip. For the micro-cantilever, active elements can be embedded into the structure to realize sensing and actuation with some of the aforementioned principles such as piezoresistivity, piezoelectricity, etc. Such active probes can help to enable new AFM capabilities with details in review [5]. For biomedical applications, selected principles can be mixed and matched based on experimental needs.

### 2.2. Nano-Positioners

A nanopositioning system regulates the relative position between the probe tip and the sample surface. Depending on the modes of operation, the deflection or oscillation characteristics of the cantilever are regulated by controlling the relative spacing between the probe tip and the sample surface. Either the probe or the sample can be scanned to create the relative motion needed for imaging.

For practical implementation, a large-range (i.e., several millimeters) coarse XYZ positioner driven by stepper motor is used to identify area of interest on the sample and engage the probe tip with the sample. A flexure-constrained scanner driven by piezoelectric actuators creates the relative to cover the scanning motion with a range over hundreds of microns at subnaometer resolution. Depending on the application needs, scanners may be exchanged to have different resolution, range and bandwidth characteristics.

### 2.3. Controller Electronics

The AFM control system is crucial to ensure good imaging performance and avoid damaging fragile biological samples. Modern AFM control systems are mostly implemented on Field Programmable Gate Array (FPGA) for the main logic. Signal conditioning circuits and driver electronics developed on custom printed circuit boards are used as the analog front end to interface with sensors and actuators. In more advanced imaging modes such as amplitude or frequency modulated dynamic modes, additional signal processing modules such as lock-in amplifiers and phase-locked loops are needed. During imaging experiments, the gains of the proportional-integral-derivative controller should be tuned by the users to ensure a good tracking performance.

### 2.4. AFM Imaging Modes

The AFM hardware can be operated in a number of modes for imaging purpose to extract various sample information. Depending on the dynamics of probe-sample interaction, they can in general be classified into four categories including contact modes, dynamic modes, jumping modes and hybrid modes.

The contact modes category is the most straightforward to understand where the cantilever deflection signal during tip-sample interaction is directly recorded or regulated during imaging. While scanning the sample, the lateral force can be measured through probe twisting to obtain friction coefficient and surface roughness information. Using a conductive probe and a biased sample, electrical properties such as voltage, current, conductance, capacitance and resistance of the sample can be measured through modes such as Scanning Voltage Microscopy (SVM), Scanning microwave microscopy (SMM), Photoconductive/Conductive Atomic Force Microscopy (PC/CAFM), Scanning Spreading Resistance Microscopy (SSRM) and scanning capacitance microscopy (SCM). The sample temperature can also be imaged with temperature-sensitive filament located near the tip through Scanning Thermal Microscopy (SThM) [6]. As the probe scans over the sample, electrical bias can be applied to excite the piezoelectric response of the material that can be measured with the cantilever deflection, giving rise to piezoresponse force microscopy (PFM). Such mechanical response can also be triggered through photothermal processes with radiation-based heating through infrared light, which corresponds to the mode of AFM-based infrared spectroscopy (AFM-IR). In addition to force responses, the optical response of the material enhanced by the plasmon effect at the probe tip and the sample surface can be utilized for Tip-Enhanced Raman Spectroscopy (TERS).

In dynamic mode AFM, the cantilever probe resonance is excited to generate a sinusoidal cantilever deflection signal during probe-sample interaction [7]. During imaging, the amplitude, phase, or frequency of this signal can be regulated to control the interaction and create contrast maps. The term “tapping mode” is often used when the interaction force is within the repulsive region of the Lennard-Jones potential between the probe tip and the sample. For the attraction region, the term “non-contact mode” AFM is used [8]. Multiple resonance frequencies can be excited simultaneously to create material property contrast mapping with more details discussed later. To measure surface potential, Kelvin Probe Force Microscopy (KPFM) technique is developed. This mode creates a topography imaging using tapping mode first and utilize the measured topography to create a repeated scan in an “interleaved” manner to characterize the surface potential and remove contribution from the topography. To measure electrostatic or magnetic force instead of surface potential, the Electrostatic Force Microscopy (EFM) and Magnetic Force Microscopy (MFM) operating in non-contact mode can be utilized. In addition to potential measurement, the cantilever tip can be utilized to enhance the localized electromagnetic field enhancement that helps to boost spatial resolution. When combined with cantilever resonance excitation, target signal with vibration signature can be extracted for spectroscopy purpose to enable the scattering-type Scanning Near-field Optical Microscopy (s-SNOM) mode. As an example in this category, the nanoscale Fourier Transformation Infrared Spectroscopy (nano-FTIR) injects dithering feature using the cantilever oscillation into the near-field signal to be distinguished from far-field noise signal using lock-in amplifiers.

In jumping mode, the resonance of the probe is not deliberately excited. Instead, the probe and the sample are brought into intermittent contact at a frequency much lower than the first cantilever resonance frequency with the peak deflection regulated using a controller. The jumping mode has many other names that can be a bit confusing, such as off-resonance dynamic mode or peak force tapping mode [9], etc. The simplest form of jumping mode is the force volume mode where a force versus distance curve is created at each location [10]. Contact mechanics model can be fitted to the obtained data to extract material property information including the snap-to-contact motion [11]. To improve the speed of imaging, Bruker company developed the patented Peak Force Tapping mode using sinusoidal approach curves during imaging. A corresponding quantitative nanomechanical property mapping mode named PFQNM is also developed. Ringing mode is an extension of the PFT mode where the residual vibration from each probe-sample interaction cycle can also be analyzed to extract additional material property information. Considering the complex interaction between soft samples and the cantilever probe tip involving adhesion, ringing mode is very helpful for nanomechanical characterization of biological samples [12]. The cantilever in this case is mostly operated in jumping mode but the residual vibration at the resonance frequency is also analyzed for imaging purpose.

Going beyond the three main categories, a number of hybrid modes exist where the tip-sample interaction can be of various nature. These including contact resonance (c-resonance) mode, force modulation mode, AFM Infrared Spectroscopy (AFM-IR), and chemical force microscopy (CFM). Contact resonance mode can be viewed as a combination of the contact modes and dynamic modes where the resonance frequency of the cantilever is excited while maintaining contact with the sample. Taking the AFM-IR mode as a more advanced example, this technique aims to characterize the photothermal absorption of the material by combining a laser source and the AFM. AFM-IR have two primary flavors including contact-mode photo-thermal induced resonance (c-PTIR) [13] and non-contact Photo-induced Force Microscopy (PiFM) [14]. A revised version for PTIR utilizes a pulsed laser at the resonance of the cantilever as resonance enhanced PTIR, which can be operated either in contact-resonance mode [15] or tapping mode [16]. For PiFM operation, the AFM cantilever piezo resonance excitation frequency and the laser pulse frequency can either operate both at the first cantilever resonance or causing deflection oscillation at both first and second resonance in a so-called sideband bimodal detection mode, which is to some extent similar to multifrequency operation. As a result, AFM-IR can be viewed as a complex hybrid mode between contact and dynamic modes.Chemical force microscopy is another hybrid mode worth mentioning. The tip of the cantilever can be functionalized with chemically active elements. The interact between the tip and the sample can be of various nature, making it a hybrid imaging mode. For biomedical applications, similar techniques have been utilized to study interaction between cells or molecules using techniques such as single cell force spectroscopy, single molecule force spectroscopy (SMFS) and single-cell force spectroscopy (SCFS).

A summary table of selected primary AFM modes of operation is provided in Table 2. It is worth to notice that researchers have also developed other operation modes for specialized application such as the pulsed-force mode [17], induced-vibration contact detection mode [18], etc. For biomedical applications, the mechanical property and spectroscopy modes are more widely used and will be discussed in more details later in this review.

## 3. AFM Biomedical Imaging Considerations

Selecting the suitable AFM modes of operation is important to ensure good imaging performance. As biomedical samples are typically soft and easily ruptured, the basic AFM contact mode imaging can deform the sample with the friction force between the probe tip and the sample during scanning. This is, in general, a more significant problem with soft biomedical samples since the sample can be damaged easily, causing severe imaging distortions. The intermittent contact between the probe tip and the sample, using tapping mode or jumping mode, is preferred for such applications.

Controlling the imaging environment properly is another important consideration to maintain biomedical sample viability. In most AFM experiments, buffered solution is used as a native environment for the samples. A fluid cell with liquid circulation can be used as the sample chamber that encloses the cantilever probe, the sample, and fluid. For more delicate samples, fluid circulation, temperature control, and carbon dioxide concentration regulation functionalities may also be included. The alignment of optical beam deflection can be more complicated even in transparent liquid due to the change of refraction index in the medium. For opaque liquid environments, a tuning-fork based AFM can conduct imaging without the transparency requirement but have a relatively high stiffness that is undesirable for biological samples [19]. For reduced stiffness, coated AFM probes with embedded active components such as a piezoresistive deflection sensor and a electrothermal actuator have been developed for imaging purposes [20].

The proper cantilever probe should be selected based on the imaging mode and operation environment. The key parameters to look for include the cantilever stiffness, first resonance frequency, tip geometry, probe, material and reflective coating material. AFM probe vendors often have recommendations for specific experiments.

For biomedical applications, optical microscope view of the sample often plays an important role as it helps to identify the area of interest. For advanced applications, fluorescence microscopy and confocal microscopy can be combined with AFM for correlative microscopy that relates images captured by both instruments. It is worth to note that a probe scan configuration is often used for AFM in this case to better match with the optical images.

**Figure 3:**
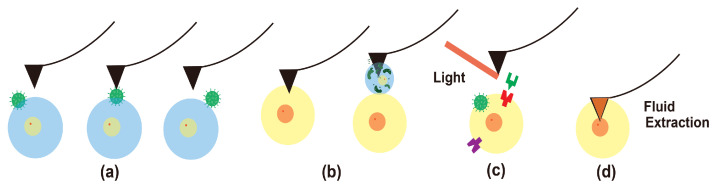
Advanced imaging capability illustration: (**a**) high-speed imaging to track virus movement on cells, (**b**) mechanobiology with viscoelastic property mapping and single-cell force spectroscopy, (**c**) chemical species characterization using near-field optics with AFM, and (**d**) AFM biomedical sample manipulation for fluid extraction from a cell.

In the following sections, the review is conducted for four advanced capabilities going beyond topography imaging in liquid, which are important in biomedical research. As illustrated in Figure 3, all four AFM capabilities have representative applications in the biomedical field.

## 4. High-Speed AFM

Improving AFM imaging speed has been a long-lasting research topic in the field. In principle, the AFM scanning process captures one pixel at a time using the probe-sample interaction. Conventional AFM imaging is a time-consuming process that takes several minutes to create even a single image. On the other hand, biological reactions can occur within milliseconds, which requires imaging at higher frame rates. For example, a skeletal myosin II power stroke is on the time scale of milliseconds, which requires high-speed AFM [21]. Due to this attractive capability to visualize dynamic processes, researchers started to improve the imaging speed since the 1990s [22] with application in biomedical research at the beginning of this century [23].

### 4.1. AFM Instrument Modification

Designing a high-speed AFM is a comprehensive engineering challenge that requires the modification of many subsystems. For the cantilever probe, nano-fabricated miniaturized cantilever probes have been developed [24,25,26]. A high resonance frequency helps the cantilever to respond more rapidly to changes of the sample topography. The first resonance frequency ω is roughly proportional to square root of the stiffness *k* to mass *m* ratio of the cantilever as $\omega \propto \frac{k}{m}$. Since the stiffness of the cantilever should remain small to avoid damaging soft biological sample, the mass of the cantilever should be reduced. The deflection sensors for miniaturized probe also needs to be modified such as a smaller laser spot in the optical beam deflection system.

For nano-positioning systems, researchers have developed a wide range of high-bandwidth scanners to improve the imaging speed [27,28,29,30]. High-bandwidth AFM scanner design is usually application-specific to optimize specific imaging parameters such as frame rate, line speed, pixel resolution, spatial resolution, range, etc. In practice, the scanner bandwidth and resolution is usually a trade of against the range of operation and need to be adjusted correspondingly. Designing scanner with better performance to push this boundary is an active field of research with techniques such as multi-actuation [31,32].

To fully realize the potential of the nano-positioners and ensure good imaging performance, high-bandwidth amplifiers and advanced control algorithms are developed. Since piezoactuators in the scanners appear as capacitive loads in the circuit, the driving electronics also need to supply large power (high current) for proper operation. Additional error-compensating capabilities can be implemented using analog circuits to remove pizeoactuator non-linearities such as hysteresis and creep [33,34,35]. Making use of the AFM scanning trajectory characteristics, advanced digital controller can be designed to improve the imaging performance. [36,37,38].

Combining these subsystem modifications, researchers have boosted AFM imaging speed from several lines per second to thousands of lines per second (tens of frames per second like a typical video) [39,40]. High-speed imaging capability allows observation of dynamic changes, which is particularly helpful for visualizing biological sample responses to external excitation by scanning over a small area (hundreds of nanometers) at tens of frames per second using HSAFM. Incorporation of HSAFM capabilities into commercial systems (e.g., JPK NanoWizard) has also made it more accessible to researchers for real-time visualization. It is worth noting that another form of high-throughput AFM can also achieve high linear scan speed with multiple probes for a large range (hundreds of microns) at a lower frame rate [41], which is suitable for inspection of static samples with a large macroscopic area over several centimeters (e.g., semiconductor wafers) instead of dynamic biomedical samples.

### 4.2. HSAFM Biomedical Applications

**Figure 4:**
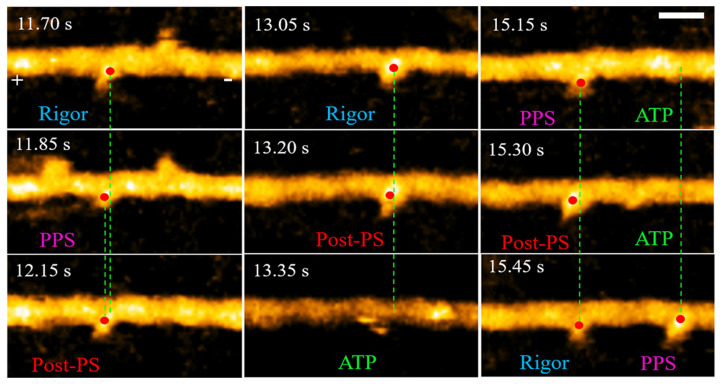
HSAFM images of skeletal HMM chemomechanical cycle: the frames show a double-headed myosin fragment bound to F-actin at different states in 2 µM ATP with the red dot indicating the center of mass. Image created at 6.7 frames per second with a 30 nm scale bar. (PPS: pre-power-stroke, post-PS: post-power-stroke) (adapted from [21] with permission, copyright 2021 American Chemical Society).

High-speed AFM imaging has enabled a variety of studies in biomedical research, especially for dynamic reactions of specimens to external stimuli. Researchers can now see the movement of DNA strands in real-time as it evolves. This is a lot more helpful than using conventional AFMs where researchers get two static images before and after a reaction without knowing what happened in between. For example, HSAFM has been used to visualize movement of a two-step motion of the double-headed heavy meromyosin (HMM) [21], as shown in Figure 4. The video is captured with 150 nm by 75 nm range with 80 by 40 pixels at 6.7 frames per second. The frames of the HSAFM video allow direct visualization of the myosin motion nanoscale resolution.

More examples of HSAFM studies in biomedical research have demonstrated the usefulness of this capability in recent years. In fundamental biological studies, HSAFM has been utilized to study cell membrane dynamics transport [42,43,44] or defects [45], DNA self-assembly [46] or damage clustering [47], RNA structure with motion [48], and the PIEZO1 mechanosensitive channel [49]. For medical applications, HSAFM has been used to study molecular dynamics of human influenza [50], avian influenza H5N1 [51], bacterial-cell interaction [52]. High-speed imaging with large scan area capability is desirable but can be very challenging, especially for soft samples. Such samples require a minimized tip-sample interaction force such that small scan areas (tens to hundreds of nanometers) are typically used in high-speed imaging. Fortunately, with a frame rate sufficiently higher than the rate of a dynamic process to be imaged, the motion of small target particles can be tracked [53]. More HSAFM imaging examples of dynamic biomolecular processes can be found in this review paper [54]. The HSAFM technique can also be combined with other techniques such as near-field fluorescence microscopy for correlative studies of biological samples [55]. As researchers get more familiarized with the HSAFM instrument, it is a promising technique to bring new understanding to the biomedical field [56].

## 5. Mechanobiology with AFM

Going beyond topography imaging, AFM can also be used to measure various material properties. As a microscopy technique that “touches” the sample surface with a physical probe, AFM is a powerful tool that measures the mechanical properties of a sample, including friction, stiffness, damping, adhesion, etc. These properties play important roles during microscopic biological interactions, including adherence, migration, proliferation, differentiation, etc. The development of cells and tissues can be tuned with mechanical signals [57]. Diseases can arise from abnormal mechanical property changes and living cells under diseased conditions such as inflammation can show changes in mechanical properties, which can be used for clinical diagnosis of cell conditions [58]. For example, as the cell mechanical properties are closely related to the progress of cancer disease and inflammation [59], AFM has been utilized for cancer cell identification and immunotherapy [60].

Different modes of AFM operation have been developed to characterize these mechanical properties. The emerging field of mechanobiology utilizes such mechanical properties to understand the physiology of cell activities and their response to mechanical excitation. The AFM modes to measure these mechanical properties primarily include Lateral Force Microscopy (LFM), force volume mode, contact resonance mode, multifrequency mode, ringing mode, and single-cell/molecule force spectroscopy. Unlike HSAFM, mechanical property mapping does not require significant modification of the instrument hardware. Adding an extra module that includes control algorithms and result interpretation to an existing AFM system would typically be sufficient to enable such new modes of operations.

Lateral Force Microscopy (LFM), which is also known as Friction Force Microscopy (FFM), is typically used for sample friction characterization. In principle, LFM is similar to contact mode AFM with an additional cantilever twisting angle measurement obtained from the laser spot horizontal movement on the quadrature photodiode. The lateral force acting on the tip is linearly proportional to this angle and can be related to the surface friction. LFM was used to study cells [61] and immunoglobulin (IgG) [62], even though it is not commonly used in biomedical research due to friction-induced sample damage.

**Figure 5:**
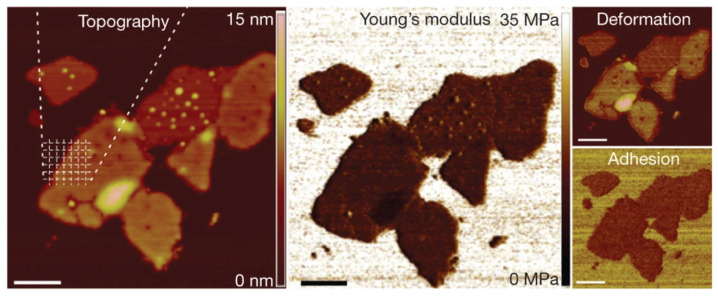
Mechanical property mapping of a native cytoplasmic purple membrane adsorbed on mica and imaged in a buffer solution with topography, Young’s modulus, deformation, and adhesion (adapted from [72] with permission, copyright 2013 Springer Nature).

The stiffness and damping parameters are considered viscoelastic properties of the sample. In its simplest form, a force-distance curve at a single location can be obtained by indenting the cantilever on the sample. The properties can be extracted through post-processing using contact mechanics models such as the Hertz model, the Johnson-Kendall-Roberts (JKR) model, the Derjaguin-Muller-Toporov (DMT) model [63], or the Maugis-Dugdale elastic contact model [64]. To create an image of sample surface properties, the force volume mode can be utilized, which basically measures multiple force curves at different locations to form a grid of hyper-pixels. Using contact mechanics models, researchers have successfully captured multiple parameters for biological samples [65] and developed algorithms for brain cancer diagnosis from force-distance curves [66]. This process can be combined with the jumping mode operation such as the Peak Force Quantitative Nanoscale Mechanical (PFQNM) mode, where a sinusoidal waveform is used for each indentation curve to improve speed [67,68,69]. The PFQNM mode has been utilized for mechanical property mapping of living cells [70]. Improving the bandwidth of this mode can also be helpful to review dynamic rate-dependent interaction as demonstrated in a high-speed cartilage rheology experiment [71]. To illustrate the mechanical property visualization capability, a multiparametric AFM image of the cytoplasmic purple-membrane surface obtained using force-distance curves and analyzed to reveal multiple parameters [72] is shown in Figure 5.

An alternative method to measure viscoelastic properties is the contact resonance mode. Based on the conventional contact mode operation, an external oscillator excites the cantilever resonance while it remains in contact with the sample surface. The resonance frequency of the cantilever-sample system increases as the sample stiffness increases, and the system’s quality factor decreases with an increase in sample damping coefficient. The resonance characteristics can be extracted by various methods such as phase-locked loop, frequency sweep, and band excitation to solve for the mechanical properties. This mode is used less frequently on soft biological samples due to friction-induced sample damage.

The multifrequency mode uses multiple resonance frequencies to create a contrast map of viscoelastic properties [73]. The mechanical AFM cantilever has, in principle, an infinite number of eigenmodes corresponding to different resonance frequencies. Since the probe-sample interaction speed and the cantilever mode shapes are different for each resonance frequency, different contrast maps can be created at each resonance frequency. In a simplified view of a lumped parameter system of the cantilever-sample system during tip-sample interaction, imaging at different resonance frequencies helps to view the viscoelastic behavior at discrete frequency points of the spectrum. Since a lock-in amplifier can extract oscillation information at a specific frequency, several cantilever resonance frequencies can be excited simultaneously during imaging to create multiple material property maps with high contrast. Cantilever geometry optimization to control eigenmode frequency difference and specialized control algorithms have been developed to improve this technique [74,75,76]. The remaining challenges and potential of multifrequency AFM for property mapping have attracted researchers to develop new machine learning and control algorithms. In biomedical studies, the multifrequency approach has been used to measure viscoelastic and rate-sensitive indentation properties of living cells [77], lipid membrane mechanical properties [78], and biomolecules [79].

Adhesion is another important mechanical property to be characterized, especially in biomedical applications. Since adhesion plays a major role during the interaction between soft cell membranes, accurately characterizing this phenomenon with an AFM can be very helpful. In its simplest form, adhesion information can be extracted from the retraction portion of the force-distance curve before the cantilever snaps out of contact from the sample surface. A recent development called the ringing mode AFM takes a step further to analyze the residual oscillation after the probe-sample detachment. This mode allows quantification of the complex adhesion phenomenon with a total of eight parameters, including the restored adhesion, adhesion height, zero-force height, disconnection height, pull-off neck size, disconnection distance, disconnection energy loss, and dynamic creep phase shift. As a relatively new imaging mode, the ringing mode has been used in mechanobiology studies to investigate multiparameter adhesion properties and create a compositional map of cells and tissues using their different properties [80].

Measuring interactions between biomolecules and cells is another important capability. Instead of using the tip-sample interaction, the probe tip can be functionalized with target biomolecules or cells to interact with the biomedical sample. Using an AFM, the Single-Cell Force Spectroscopy (SCFS) or Single-Molecule Force Spectroscopy (SMFS) modes can be utilized to measure interactions and binding forces between molecules or cells. In these modes, the probe tip or a tip-less cantilever can be functionalized with a specific molecule or a cell. By pressing the functionalized probe onto target objects, the interaction force can be quantified with the cantilever deflection measurement. In biomedical applications, the binding force and energy of biomolecules (e.g., protein) can be measured using this method to understand the nanoscopic interaction between cells such as kidney cells [81], red blood cells [82], cancer cell identification [83] and probing native membrane proteins [84]. Such measurements can also be conducted with optical or magnetic tweezers, which offers fewer imaging modalities but easier cell manipulation capability than conventional AFM tip functionalization [85]. However, with the development of FluidFM technology, to be discussed later, AFM can also pick up cells using microfluidic techniques to conduct force spectroscopy [86], making the AFM a more versatile tool for such applications.

## 6. AFM Molecular Species Characterization

Molecular chemical species identification is typically conducted using spectroscopy techniques. Spectroscopy technology utilizes the interaction between material and electromagnetic radiation at various frequencies. By collecting the information of input radiation signal for the source and output radiation signal after reaction with the sample, a spectrum plot of the signal of interest can be produced. As different materials or chemical bonds can react differently to radiation at distinct wavelength, spectroscopy are widely used to identify the presence of certain elements and bonds. Raman spectroscopy and Infrared Spectroscopy are two main types of spectroscopy methods. Raman spectroscopy utilize incident light to excite molecules that emits light in a different wavelength. On the other hand, infrared spectroscopy is relies on the sample absorption of infrared light.

The infrared regime is an extremely important spectrum range of electromagnetic waves. The corresponding energy scale ( 50 meV to 1 eV) coincides with many physical processes that happen in solid materials and molecules, such as the free electron conductivity in metals, interband transitions in narrow bandgap semiconductors, lattice phonon vibrations in crystals, and vibration modes in molecules. The most popular spectroscopy tools in the infrared spectrum is the Fourier Transformed Infrared Spectroscopy. Utilizing a broadband laser source and a Michelson interferometer setup to collect experimental data over a wide spectral range as opposed to dispersive spectrometers that collects data at a very narrow band gap. Being able to obtain microscopy and spectroscopy information on materials in the infrared spectrum range can lead to numerous scientific and technological applications such as molecular spectroscopy, thermal imaging, material analysis and biomedical imaging. The typical spectral resolution of such instrument is 0.5 cm${}^{-1}$ while resolution as high as 0.001 cm${}^{-1}$ has also been achieved before [87]. While the high-resolution spectrum information is obtained with FTIR, its spatial resolution remains limited due to the beam spot size. The spatial resolution for contrast between various material is typically on the order of several microns.

### 6.1. Tip-Enhanced Scanning Near-Field Optical Microscopy

Scanning Near-field Optical Microscopy (SNOM) is used to overcome the far field spatial resolution limit of beam spot by the Rayleigh criteria. Making use of evanescent waves in the near-field, higher spatial resolution images can be obtained with AFMs operated either with or without a probe tip aperture. In the aperture mode, the light emitted and collected is confined in an aperture on the probe tip with size smaller than the wavelength for high spatial resolution spectrum information collection. In aperture free modes, it is important to distinguish the near-field target signals from the far-field noise signals. This is relatively easy for Raman spectroscopy since its excitation and response wavelengths are different. For the infrared spectrum, dithering signal from tapping mode AFM probe tip is utilized to modulate the near-field signal with sinusoidal waveforms, which helps to extract the near-field signal using a lock-in amplifier. When operating at a single location, the spatial resolution of scanning probe enhanced spectroscopy techniques is significantly improved.

It is worth to note that SNOM imaging using infrared wavelength is in principle different from AFM-IR. Although both techniques make use of the difference in absorption spectrum of the material, AFM-IR relies on the heating effect of the absorbed laser to induce mechanical expansion of the material.

To create a map for density of specific elements or bonds, the spectrum magnitude or phase at the corresponding characteristic wavelength in the spectrum can be extracted to form a single point of the map. By scanning the incident light spot over the sample surface, an intensity plot can be created to form a density map [88,89]. For more demanding applications where the spectrum is desired at each spatial location, hyper-pixels can be collected for further analysis.

To enable SNOM capabilities, optical components need to be coupled into the AFM system. The modification to the AFM system is relatively small but a relatively large area for access to the probe and sample is desired. For custom designs, commercial AFMs with a relatively large opening for light coupling is preferred as a baseline system for topography imaging (e.g., Bruker Innova). Light path for the laser source and corresponding detectors can be coupled into the system to extract the desired signal. As the metal-coated AFM probe tip enhances the near-field signal, its dimension should be comparable to the wavelength of the incident laser. The scattering type SNOM incident laser typically has an angle of 30 degrees with respect to the in-plane sample surface to have a large projected area on the sample without being blocked significantly by the cantilever. Moreover, a smaller focused spot of the laser is desirable to concentrate the energy to the probe tip for improved signal to noise ratio. To realize this, laser beam with good collimation and large beam waist diameter should be used before it is focused to the probe tip using a lens or a parabolic mirror, which is typically achieved using optical beam expanders.

### 6.2. AFM Molecular Spectroscopy Biomedical Applications

Identifying molecular species of a biomedical samples is another important capability for advanced studies. Optical spectroscopy techniques can be integrated with AFM systems to realize this desired capability based on different spectrums of chemical bonds. In this case, the AFM tip helps to achieve super-resolution down to tens of nanometers [90] for the optical signal by making use of near-field optics, giving rise to the Scanning Near-field Optical Microscopy (SNOM). As the AFM keeps track of the sample topography, the optical signal is collected simultaneously. In its early stage of development, a coaxial scanning setup is utilized with an aperture through the cantilever probe tip. The laser beam is confined in the channel and the laser reflected or transmitted through a relatively thin sample is captured. In this setup, the near-field signal spatial resolution depends on the size of the aperture. As an alternative, a scattering-type SNOM has been developed where no aperture is needed on the probe tip. In scattering-type SNOM, the laser beam is focused on the tip-sample interaction point and the cantilever probe operates in tapping mode at resonance, which injects a dithering signal that modulates the near-field component of the laser. The near-field signal can be extracted from the far-field using a lock-in amplifier from the scattered reflected light. Due to the non-linear near-field intensity to probe-sample separation distance relation, the lock-in frequency can be set to integer multiples of the tapping dithering signal for an improved signal-to-noise ratio.

Depending on the chemical species to be identified, different types of spectroscopy techniques can be utilized. For example, Tip Enhanced Raman Spectroscopy (TERS) and nanoscale Fourier Transformation Infrared Spectroscopy (nano-FTIR) can be used to identify chemical species from the spectrum with a high spatial resolution. Some materials can also generate heat-induced force with light absorption, giving rise to another technique called photo-induced force microscopy. Depending on the underlining physical phenomenon, different imaging modes can be selected to identify the chemical species.

**Figure 6:**
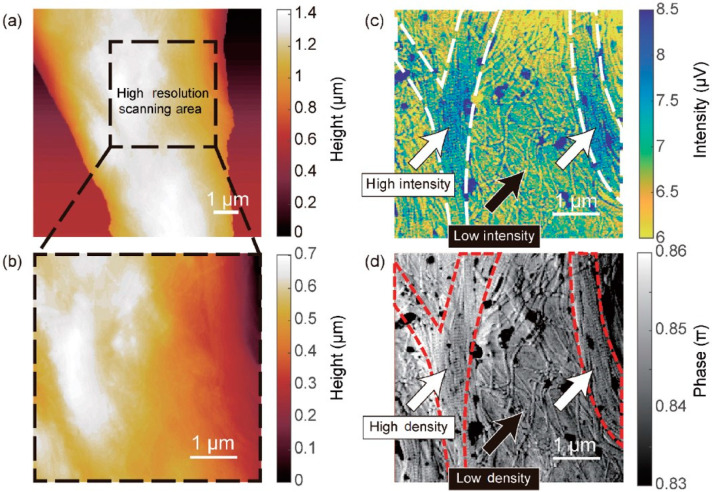
Scattering type SNOM image of collagen fibrils on a nerve section surface: (**a**,**b**) topography image of a section of the collagen fibrils, (**c**) third harmonic amplitude image at 1100 cm${}^{-1}$ with labels of the high-density area, (**d**) force phase image of the collagen fibrils (adapted with permission from [91], copyright 2021 Springer Nature).

For biomedical applications, revealing chemical species can help to review hidden information previously unobservable through topography images. As an example, a recent study investigates the sliding dislocation of collagen fibrils, whose arrangement defects can play an important role in many common diseases such as bone fracture and fibrosis [91]. Using the scattering-type SNOM techniques as shown in Figure 6, two distinct collagen fibril arrangements are reviewed, which helps to establish a deeper understanding of collagen-related diseases. It is worth noting that the phase image from tapping mode operation also reveals material mechanical property contrast that to some extent matches with the SNOM image, which further confirms the existence of different material structures.

In fundamental biological research, scattering-type near-field techniques have been used widely to study protein [92,93], cells [94], phospholipid bilayers [95], virus [96], and membrane [97]. For medical applications, SNOM has been used for human hair sample study [98] and cancer cell detection [99]. As a new powerful technique with hype-pixel spectrum at each location, data-driven algorithms could be a powerful technique to uncover hidden information from a large amount of data. Researchers have started to create datasets to enable the training of algorithms such as the dataset for bacterial cells [100]. In addition to infrared s-SNOM, AFM-IR technique has also been used in biomedical applications. As an example, label-free detection of biotoxins have been realized with PiFM, a technique beloning to the AFM-IR category as discussed previously [101]. The material property mapping technique can also be combined with other techniques, such as fluorescence microscopy [102] and Raman imaging [96], for in-situ correlative microscopy [103] to reveal more sample details.

## 7. Probe-Based Nano-Manipulation and Nanofabrication

In addition to its conventional use as a characterization tool, AFM can be a powerful nano-manipulation tool with atomic resolution [104,105]. During imaging, the tip-sample interaction force should be minimized to avoid affecting the sample being measured. On the other hand, for manipulation applications, the interaction force is deliberately increased in a controlled manner to augment the sample surface.

### 7.1. AFM Sample Surface Modification

AFM-based sample surface modification can in general be classified into three categories including lithograhpy (existing surface modification), deposition (addition of extra material) and manipulation (moving objects).

In its simplest form, AFM tips can create patterns on the sample surface. By increasing the set-point force directly proportional to the cantilever deflection, Mechanical scanning probe lithography (M-SPL) can create microscopic scratches on the sample surface for material removal [106]. To assist pattern creation on the surface, various conditions can be applied to induce reactions including field-emission SPL, dip-pen SPL, oxidation SPL, thermal SPL and thermochemical SPL. In field-emission SPL, electrical bias is applied to modify the resist property to be either negative or positive tone depending on the dosage to create patterns [107]. In dip-pen lithography, AFM probe coated with specialized “ink” is used to etch the substrate through electrostatic or electrochemical interactions. In principle, it can also be used to add small amount of material to the substrate surface. For oxidation, thermal and thermomechanical SPL, anodizing or thermochemical reactions between the probe and the substrate surface are utilized for pattern creation. A detailed review of such process can be found in [108]. A summary of AFM-based surface modification capabilities classified into these three categories is provided in Table 3 with references to representative work in the field.

For material deposition, AFM can be used as a nanoscale 3D printer with material fed through an aperture near the cantilever probe tip. The small size aperture allows position control with high spatial resolution during deposition. For material processing in vacuum, atomic scale single-ion implantation can dope selected area on the substrate [120]. In the same manner, a particle jetting system have been developed to precisely deposit nanoparticles on the substrate [115]. For liquid operation more relevant to biomedical applications, the fluid force microscopy technique utilizes a hollow cantilever with an aperture at the tip to locally dispense materials via pressure control. Notice that similar SPM techniques such as Scanning Ion Conductance Microscopy (SICM) and Scanning ElectroChemical Microscopy (SECM) utilizing sharpened pipette tip can also deposit material locally in liquid with a simpler probe fabrication process. However, SICM in-situ topography imaging of the sample requires AC excitation and conductive liquids, which is more restricting compared to an AFM setup.

For manipulation of the substrate surface, STM can routinely achieve atomic resolution imaging and atom manipulation by controlling the bias voltage. Researchers have used AFM probe tip for pick & place manipulation of ions such as Br${}^{-}$ on NaCl lattice structure [117]. On the larger scale, AFM cantilever probe can be used to move and rotate samples such as 2D material flakes in a controlled manner [119]. Moving on to an even larger scale, fluidFM system can be used to pick and place micro-meter scale cells by applying proper suction force through the pressure control system to capture and release the target cells. Moreover, the fluidFM system can be used to sample biological substances via the orifice on the probe tip.

Modification to the existing AFM system to enable nanoscale lithography, deposition and manipulation primarily centers around cantilever probe tip functionalization and material preparation. The modification to AFM hardware systems is less significant compared to high-speed imaging and can be relatively easily incorporated into existing commercial AFM systems.

### 7.2. AFM Biomedical Sample Manipulation

**Figure 7:**
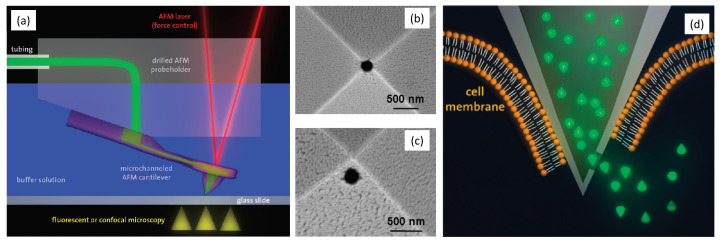
FluidFM principle illustration: (**a**) schematic diagram of a hollow cantilever with microfluidic channels, (**b**,**c**) SEM image of a FluidFM probe tip with openings on the tip apex for cell manipulation and near the tip apex for particle injection and sampling, and (**d**) schematic of an injection process of particles into cells after penetrating the membrane (adapted with permission from [121], copyright 2009 American Chemical Society).

For biomedical applications in liquid environments, the FluidFM has become a particularly useful tool since its invention in 2009 [121]. Using a hollow cantilever with an opening near the tip, as shown in Figure 7, an external microfluidic system can be utilized to apply suction pressure for the pick-and-place manipulation of cells. Such manipulation capabilities allow researchers to modify biomedical samples on the nanoscopic scale, which is much more precise compared to conventional needle-based micro-manipulation systems. This is a particularly convenient capability for cellular biophysics studies [122], such as cellular adhesive interactions [123,124], stress-dependent yeast cell mating [125], and cellular detachment forces and energies [126].

As a second capability, if the probe tip creates small openings on the cell by penetrates through the membrane, liquid can also be injected into [118] or extracted from [127,128,129] the cell through the orifice on the cantilever tip with precise volume control. Last but not least, the hollow cantilever can be used for local dispensing of liquid to create patterns on the sample surface. This allows precise control of molecular assembly [130], 2D patterning [131], nanoscale 3D printing [114,132], and 3D lithography [133]. FluidFM techniques have started to become a popular cell manipulation tool in addition to optical and magnetic tweezers.

## 8. Outlook and Future Perspectives

### 8.1. Additional Modes and Applications

A good historical review of advanced AFM capability developments based on experimental needs can be found in reference [134]. In addition to the aforementioned capabilities, researchers have also used AFM to explore some less common biomedical applications. For example, electrical signals and properties in biological samples can be studied using modes such as Electrostatic Force Microscopy (EFM), Kelvin Probe Force Microscopy (KPFM), and Conductive Atomic Force Microscopy (CAFM) as reviewed in reference [135].

AFMs have been used in several practical medical applications. Many pathologcial conditions have been investigated with AFM including articular cartilage and osteoarthritis, cancer, Alzheimer’s Disease, and viruses such as HIV [136]. More examples include identifying novel drug targets in drug discovery [137], imaging collagen fibrils of the cornea in vision science [138], characterization of internal mammary artery [139], and dental studies of fluoride treatment on phosphoric acid-etching in primary teeth [140]. As the reviewed imaging capabilities become more matured and commercialized, more research studies in the biomedical area are expected to make use of AFM as a powerful tool. Since this review is conducted mainly from an instrumentation perspective, an application perspective can be helpful for interested readers as in [136].

For biomedical research, functionalization of the AFM probe tip can greatly extend the range of applications. Just as single molecular force spectroscopy discussed previously, attaching functional elements to the probe tip allows richer interactions to be investigated. Research efforts in this direction would require profound knowledge about the sample which is beyond the expertise of instrumentalists. As a result, collaborative research to identify the desired functionalization and realization of such probes would be helpful.

### 8.2. AFM Instrumentation

Extension of AFM capabilities is an on-going effort to meet the evolving demands of industrial applications and scientific research. For example, using parallel active cantilever arrays and image stitching, researchers have developed AFM system for large samples. Combining nanoscale resolution and centimeter scale imaging range makes this tool ideal for industrial applications such as semiconductor wafers. This technique can potentially be helpful for inspection of tissue samples over a large area to obtain statistical information about macroscopic organisms.

For the future development of instrument capabilities, merging multiple imaging modalities can be helpful. Correlative imaging of biomedical samples using optical microscopy and AFM is a helpful technique where fluorescence markers can be used to identify the object of interest. Techniques such as second harmonic generation and SEM can also be colocalized with AFM to investigate the same area of the sample. Following similar philosophy, merging of different AFM characterization capabilities into the same instrument can be helpful for advanced research investigation. For example, if high-speed AFM topography imaging can be extended to include modalities such as SNOM or nanomechanics, real time property variations of the sample in response to changes of external environments can be probed. In summary, development of new imaging modalities and merging of various capabilities can both help to lead to new fundamental discoveries.

## 9. Conclusions

In this paper, key advanced AFM capabilities relevant to biomedical applications are reviewed. Going beyond the basic topography imaging, advanced capabilities are discussed, including high-speed imaging, mechanical property mapping, chemical species identification, and nano-manipulation/fabrication. By introducing principles of relevant modes with recent applications in biomedical studies, this review can help biomedical and pharmaceutical researchers get familiarized with the AFM as a powerful research tool.

Many of the advanced AFM modes have been developed based on application needs by instrumentalists or researchers who have specific experimental needs. The AFM field is also continuously evolving to enable new functionalities. Moreover, combining multiple imaging capabilities into the same system would also extend AFM application scenarios. As an example discussed, obtaining high-speed imaging with chemical species mapping simultaneously would allow better visualization of biological reaction processes. With the increasing research demands, we believe that more capabilities will arise from the active AFM research community, which will, in turn, benefit the biomedical research communities.

## Figures and Tables

**Table 1:** Comparison of Main Microscopy Techniques.

Microscopy	Optical	SEM	TEM	AFM	STM
Resolution	200 nm	10 nm	0.2 nm	2 nm	0.1 nm
Typical Image Size	1000 μm	1000 μm	100 μm	100 μm	0.5 μm
Typical Frame Rate	100 FPS	20 FPS	20 FPS	0.1 FPS	0.1 FPS
Main Modality	2D image	3D image	2D projection	3D Topography	3D density of states
Environment	vacuum, air, liquid	vacuum	vacuum	vacuum, air, liquid	vacuum, air

**Table 2:** AFM Imaging Mode Summary.

Category	Mode Name	Modalities	Modification	Main Benefits	Main Limitations
Contact	Constant height	topography	no *z* control	simple control & high-speed	changing force, lateral friction
	Constant force	topography	standard *z* PID	easy high-speed	lateral friction
	Lateral force	friction	twist detection	roughness	sample scratching
	PFM	piezoelectric	electrical bias	Domain identification	lateral friction
	SMM	impedance	network analyzer	versatile samples	lateral friction
	SVM	voltage	conductive path	voltage measurement	conductive probe wear
	PC/CAFM	current	conductive path	current measurement	conductive probe wear
	SSRM	resistance	conductive path	resistance measurement	sample property spreading
	SCM	capacitance	conductive path	capacitance measurement	ambient water meniscus trap
	SThM	temperature	thermal filament	temperature mapping	filament-tip offset
	TERS	Raman	optical parts	chemical species mapping	laser focusing overhead
Dynamic	AM tapping	topography	lock-in amplifier	amplitude control	slower imaging
	PM tapping	topography	lock-in amplifier	phase control	slower imaging
	FM Tapping	topography	phase-locked loop	Quality factor robustness	frequency instability
	Non-contact	topography	lock-in amplifier	minimal sample damage	ambient water meniscus trap
	Multifrequency	nanomechanics	lock-in amplifier	stiffness, damping, etc.	available cantilever resonance
	KPFM	surface potential	conductive path	chemical potential mapping	slow operation
	EFM	electrostatic	conductive path	electrostatic force	ambient water meniscus trap
	MFM	magnetic	magnetized tip	magnetic force	stray magnetic field effect
	s-SNOM	spectroscopy	optical parts	chemical species spectroscopy	light path access
Jumping	Force volume	nanomechanics	algorithm	full indentation curve	slower imaging
	Peak force	topography	algorithm	easy experiment setup	proprietary technology
	PFQNM	nanomechanics	algorithm	easy quantitative mechanics	proprietary technology
	Ringing	nanomechanics	algorithm	stiffness, adhesion, etc.	proprietary technology
Hybrid	C-resonance	nanomechanics	algorithm	stiffness, damping, etc.	sample scratching
	AFM-IR	photothermal	light source	spectroscopy contrast	absorption rate
	CFM	chemical forces	probe tip functionalization	chemical species interaction	tip/sample preparation

**Table 3:** AFM Manipulation and Nanofabrication Capability Summary.

Category	Principles	Modification	Application Examples	Reference
lithography	mechanical	larger interaction force	pattern on nanophotonic devices	[109]
	thermal	heatable probe tip	low-force data storage patterning	[110]
	thermo-chemical	heatable probe, reactions	protein gradient patterning	[111]
	oxidation	heated probe, reactions	nano-wire transistors	[112]
	dip-pen	positioning control sequence	electrostatic/chemical patterning	[113]
	field-emission	controlled electrical bias	quantum dot transistors	[107]
Deposition	fluidFM printing	hollow cantilever & tip aperture	3D metal printing, self-assembly	[114]
	nanojet	particle system & tip aperture	local particle placement	[115]
	ion implantation	ion source & tip aperture	local-doping on silicon	[116]
manipulation	pick & place	functionalized tip	fix atomic defects	[117]
	inject & sample	pressure system & hollow tip	collect cell substances	[118]
	move & twist	flexible motion control system	stacking 2D material flakes	[119]

## Abbreviations

The following abbreviations are used in this manuscript:

AFM	Atomic force microscopy
SPM	Scanning probe microscopy
STM	Scanning tunneling microscopy
TERS	Tip-enhanced Raman spectroscopy
SVM	Scanning voltage microscopy
SThM	Scanning thermal microscopy
PiFM	Photo-induced Force Microscopy
PC/CAFM	Photoconductive/Conductive Atomic Force Microscopy
SSRM	Scanning spreading resistance microscopy
AM	Amplitud modulation
PM	Phase modulation
FM	Frequency modulation
KPFM	Kelvin probe force microscopy
PFT	Peak force tapping
PFM	Piezoresponse force microscopy
PFQNM	Peak force quantitative nanomechanics
SCM	Scanning capacitance microscopy
SMM	Scanning microwave microscopy
EFM	Electrostatic force microscopy
MFM	Magnetic Force Microscopy
CFM	Chemical force microscopy
c-PTIR	Contact-mode photo-thermal induced resonance
s-SNOM	Scattering-type scanning near-field optical microscopy
nano-FTIR	Nanoscale Fourier Transformation Infrared Spectroscopy
FluidFM	Fluid force microscopy
SPL	Scanning probe lithography
SICM	Scanning Ion Conductance microscopy
SECM	Scanning electrochemical microscopy
LFM	Lateral force microscopy
FFM	Friction force microscopy
HSAFM	High-speed atomic force microscopy
SMFS	Single molecule force spectroscopy
SCFS	Single-cell force spectroscopy
